# German translation, cross-cultural adaptation and validation of the whiplash disability questionnaire

**DOI:** 10.1186/1477-7525-11-45

**Published:** 2013-03-14

**Authors:** Corina Schuster, Michael McCaskey, Thierry Ettlin

**Affiliations:** 1 Research Department Reha Rheinfelden, Salinenstrasse 98, 4310 Rheinfelden, Switzerland; 2 Department of Engineering and Information Technology, Institute for Rehabilitation and Performance Technology, Bern University of Applied Sciences, Bern, Switzerland; 3 Department of Health Sciences and Technology, Institute for Human Movement Sciences, ETH Zurich, Zurich, Switzerland; 4 Department of Behavioural Neurology, Medical Faculty, University of Basel, Basel, Switzerland

**Keywords:** Whiplash injury, Questionnaire, Impairment, Pain, Activities of daily living, Kraniozervikales beschleunigungstrauma, Fragebogen, Schmerz, Einschränkungen, Aktivitäten des täglichen Lebens

## Abstract

**Background:**

The Australian Whiplash Disability Questionnaire (WDQ) was cross-culturally translated, adapted, and tested for validity to be used in German-speaking patients. The self-administered questionnaire evaluates actual pain intensity, problems in personal care, role performance, sleep disturbances, tiredness, social and leisure activities, emotional and concentration impairments with 13 questions rated on an 11-point rating scale from zero to ten.

**Methods:**

In a first part, the Australian-based WDQ was forward and backward translated. In a consensus conference with all translators and health care professionals, who were experts in the treatment of patients with a whiplash associated disorder (WAD), formulations were refined. Original authors were contacted for clarification and approval of the forward-backward translated version. The German version (WDQ-G) was evaluated for comprehensiveness and clarity in a pre-study patient survey by a random sample of German-speaking patients after WAD and four healthy twelve to thirteen year old teenagers.

In a second part, the WDQ-G was evaluated in a patient validation study including patients affected by a WAD. Inpatients had to complete the WDQ-G, the North American Spine Society questionnaire (NASS cervical pain), and the Medical Outcomes Study 36-Item Short Form Health Survey (SF-36) at entry in the rehabilitation centre.

**Results:**

In the pre-study patient survey (response rate 31%) patients rated clarity for title 9.6 ± 0.9, instruction 9.3 ± 1.4 and questions 9.6 ± 0.7, and comprehensiveness for title 9.6 ± 0.7, instruction 9.3 ± 1.4 and questions 9.8 ± 0.4. Time needed to fill in was 13.7 ± 9.0 minutes.

In total, 70 patients (47 females, age = 43.4 ± 12.5 years, time since injury: 1.5 ± 2.6 years) were included in the validation study. WDQ-G total score was 74.0 ± 21.3 points (range between 15 and 117 points). Time needed to fill in was 6.7 ± 3.4 minutes with data from 22 patients. Internal consistency was confirmed with Cronbachs’s α = 0.89. Concurrent validity showed a highly significant correlation with subscale pain and disability (NASS) at r = 0.74 and subscale pain (SF-36) at r = 0.71.

**Conclusions:**

The officially translated and adapted WDQ-G can be used in German-speaking patients affected by a WAD to evaluate patients’ impairments in different domains. The WDQ-G is a self-administered outcome measure showing a high internal consistency and good concurrent validity.

## Abstract (de)

**German abstract:**

**Hintergrund**

Der australische Whiplash Disability Questionnaire (WDQ) wurde für deutschsprachige Patienten übersetzt, angepasst und auf seine kriteriumsbegzogene Validität getestet. Der WDQ ist ein selbstauszufüllender Fragebogen mit 13 Fragen bezüglich Schmerz, Einschränkungen in der persönlichen Pflege, Rollenerfüllung, Schlafstörungen, Erschöpfung, soziale und Freizeitaktivitäten, emotionale und Konzentrationsprobleme, die auf einer Skala von null bis zehn bewertet werden.

**Methoden**

In einem ersten Teil wurde der aus Australien stammende WDQ vorwärts und rückwärts übersetzt. In einer Konsensuskonferenz mit allen Übersetzern, Therapeuten und Ärzten mit Erfahrung in der Behandlung von Patienten nach einem Kraniozervikalen Beschleunigungstrauma (KZBT) wurden widersprüchliche Formulierungen angepasst und verfeinert. Danach wurden die Originalautoren für die Prüfung der vorwärts-rückwärts übersetzten Versionen und deren Einsatz am Patient kontaktiert. In einer Vorstudie wurde die deutsche Version des WDQ (WDQ-G) auf Klarheit und Verständlichkeit in einer Zufallsstichprobe von 47 deutschsprachigen Patienten nach einem KZBT und vier zwölf- bis 13-jährigen Jugendliche und überprüft.

In einem zweiten Teil (Validierungsstudie) füllten Patienten nach einem KZBT bei ihrem stationären Eintritt den WDQ, den North American Spine Society Fragebogen (NASS zervikaler Schmerz) und den Fragebogen der Medical Outcomes Study 36-Item Short Form Health Survey (SF-36) aus.

**Resultate**

In der Vorstudie (Rücklaufquote 31%) bewerteten die Patienten die Klarheit für Titel 9.6 ± 0.9, Instruktion 9.3 ± 1.4 und Fragen 9.6 ± 0.7, sowie Verständlichkeit für Titel 9.6 ± 0.7, Instruktion 9.3 ± 1.4 und Fragen 9.8 ± 0.4. Die Zeit zum Beantworten der 13 Fragen betrug 13.7 ± 9.0 Minuten.

In die Validierungsstichprobe wurden 70 Patienten (47 Frauen, Durchschnittsalter = 43.4 ± 12.5 Jahre, durchschnittliche Zeit seit Ereignis 1.5 ± 2.6 Jahre) eingeschlossen. Der WDQ-G Totalwert betrug 74.0 ± 21.3 Punkte (Median 75.5 Punkte, Streuweite zwischen 15 und 117 Punkten). Die Zeit zum Ausfüllen des WDQ-G wurde mit 6.7 ± 3.4 Minuten für die Validierungsstichprobe mit Daten von 22 Patienten berechnet. Die interne Konsistenz wurde mit Cronbach’s α = 0.89 bestätigt. Die kriteriumsbezogene Validität zeigte eine hochsignifikante Korrelation mit der Subskala Schmerz und Einschränkung (NASS) r = 0.74 und der Subskala Schmerz (SF-36) r = 0.71.

**Konklusion**

Der offiziell übersetzte und angepasste WDQ-G kann bei Patienten nach einem KZBT eingesetzt werden, um Einschränkungen in verschiedenen Domänen einzuschätzen. Der WDQ-G ist ein selbst auszufüllender Fragebogen, welcher eine hohe interne Konsistenz und eine gute kriteriumsbegzogene Validität aufweist.

## Background

Neck pain can pose a substantial limitation in daily life and profession for affected individuals and family members. Globally, about 180 of 1000 people experience neck pain at least one day a year [1]. In 1995 the Québec Task Force on Whiplash Associated Disorders (WAD) defined the disorder as “an acceleration-deceleration mechanism of energy transferred to the neck that results in soft tissue injury that may lead to a variety of clinical symptoms.” [2]. That mechanism can occur predominantly in motor traffic accidents but also in injuries related to sport and work [3]. Holm and colleagues determined an annual incidence of at least 300 per 100,000 inhabitants for North America and Western Europe [3].

Guzman *et al.* introduced a new conceptual model of neck pain based on the work of the Task Force on Neck Pain and Associated Disorders during the Bone and Joint Decade 2000 to 2010 [4]. The model can be applied to different disorders and causes of neck pain including WAD. Developed on the literature found in a systematic search between 1980 and 2006 the model included five components describing risk factors for pain development, its re-occurring character, pain onset and course, pain management, and the impact of pain on life. In particular, the impact of pain on life should be evaluated with a specific questionnaire. This was addressed by the Neck Disability Index (NDI) and the Northwick Park Neck Pain Questionnaire (NPQ) [5,6]. In a cross-sectional comparison study the NDI and the NPQ have been investigated in patients with WAD [7]. Participating patients identified seven categories that were either only evaluated by the NDI or the NPQ. None of the investigated questionnaires could cover all WAD-specific categories, e.g. emotional and social aspects. Results of Hoving *et al.*’s evaluation highlights the need for a disease-specific questionnaire. Therefore, the Whiplash Disability Questionnaire (WDQ) was specifically developed for individuals suffering from a WAD by Pinfold and colleagues and published in 2004 [8]. The development of the original WDQ comprised four steps: a) item generation based on the existing NDI items and semi-structured interviews with 83 patients from Hoving *et al.*’s study [7], b) preliminary clinical testing with 101 patients, and c) expert review of the developed questionnaire. The WDQ is a disease-specific self-administered outcome measure to evaluate pain intensity and limitations due to a WAD in different domains: present pain levels, personal care, role performance, mobility, sleep disturbances, tiredness, social and leisure (sporting and non-sporting) activity, emotional and cognitive impairments.

Two systematic literature reviews on neck pain questionnaires critically appraised their quality and availability in different languages [9,10]. Authors found no publication on a German WAD-specific questionnaire but they found two publications on a German version of the Neck Pain and Disability Scale to evaluate German-speaking patients with non-specific neck pain or neck pain related to fusion surgery (C1-C2) [11,12]. The translation process described in both publications was classified as fair to poor [9]. No information on responsiveness of the German versions could be obtained. So far, no WAD-specific questionnaire exists that could be used in German-speaking individuals offering a trustworthy translation procedure and quality criteria.

Currently, the officially translated North American Spine Society questionnaire (NASS) and the Medical Outcomes Study 36-Item Short Form Health Survey (SF-36) have been used to evaluate treatment effects of patients with neck pain [13-21]. Consequently, the aims of the present study were formulated as follows:

1) to establish a German version of the WDQ following recommended guidelines,

2) to test the concurrent validity with the subscale pain and disability of the German NASS for cervical spine and with the subscale bodily pain of the German SF-36, and

3) to examine internal consistency of the German WDQ.

It is hypothesised that a German WDQ will highly correlate with the subscale pain and disability of the German version of the NASS for cervical spine and with the subscale bodily pain of the German version of the SF-36.

## Methods

### First part: translation and adaptation process

Translation and trans-cultural adaptation guidelines for self-administered outcome measures of Beaton *et al.* (2000) [22] was used as basis for the procedure applied in this study. The guidelines include six stages:

#### Stage (1) Translation into the target language

Three German speaking translators produced independent forward translations of the WDQ. One was done by an officially recognised translator with a history of low back pain, the second one by an English teacher, who experienced a WAD, the third one by a physiotherapist, who is specialised in neurological rehabilitation and has worked in an English speaking country for several years.

#### Stage (2) Synthesis of the forward translations

Forward translations were synthesised into one German version by the project leader.

#### Stage (3) Backward tanslations

The synthesised forward translated version was then backward translated into English by three independent translators: a bilingual physician, a bilingual financial analyst, and an English native-speaking housewife living in the German part of Switzerland for more than ten years. The backward translations were again synthesised by the project leader.

#### Stage (4) Consensus conference

In a two-hour long consensus conference all forward and backward translators, two occupational therapists, an additional physiotherapist, an additional physician, and the project leader reviewed the synthesised forward translated German and the backward translated English version. All healthcare professionals were experts with experiences in the treatment of patients with a WAD. A consensus version was produced, representing the preliminary German version of the WDQ, termed WDQ-G. The conference lasted for about two hours.

#### Stage (5) Pre-study patient survey

A randomly selected sample of former inpatients (60 out of 1019 patients between 1999 and 2005 of a Swiss rehab centre) diagnosed with WAD received the preliminary WDQ-G version by postal mail. Patients were asked to fill in the questionnaire and rate clarity and comprehensiveness of the title, questionnaire instruction, and questions on two eleven-point visual analogue scales (VAS), ranging from zero to ten (where ten indicated the highest level). Furthermore, all patients were asked to report the time needed to fill in the preliminary WDQ-G. The preliminary WDQ-G was moreover rated by four healthy twelve to thirteen year old teenagers for clarity and comprehensiveness.

#### Stage (6) Approval of original authors

The preliminary WDQ-G and all forward and backward translations were sent to the original authors, asking for approval to use the WDQ-G in a patient validation study.

### Second part: patient validation study

#### Study design

After receiving permission from the original authors to use the pre-final WDQ-G, a patient validation study was carried out. Inpatients were asked to fill in the questionnaires at entry. The study was approved by the responsible ethics committee in Aarau (reference number: 2005/039) and carried out in accordance with the Declaration of Helsinki.

#### Outcome measures

Based on the synthesis report by Frinking *et al.*, data on influential factors (e.g. time since injury, insurance status, employability, number of comorbidities, and related treatments and impairments, medication, and demographic data (e.g. age, gender) were collected from each patient [23].

Patients completed the preliminary WDQ-G, one cervical problem-specific questionnaire and two generic instruments, which had been validated in several previous studies [16,24-29]. All questionnaires used will be described below.

##### The Whiplash Disability Questionnaire (WDQ)

The WDQ has been developed based on items of the existing NDI and semi-structured interviews conducted with 83 patients by Hoving and colleagues [7]. Patients have emphasised not to focus on present pain level but also to evaluate further domains that might be affected by a WAD, e.g. personal care, role performance, mobility, sleep disturbances, tiredness, social and leisure (sporting and non-sporting) activity, emotional and cognitive impairments [8]. Each of the 13 questions is rated on an 11-point scale ranging from zero to ten. The total score can vary between zero and 130 points. A high total score indicates a high level of perceived impairment. It takes about five to ten minutes to fill in the WDQ and does not require specific training [30]. In the present study, patients were asked to fill in the preliminary WDQ-G.

##### The North American Spine Societies Questionnaire (NASS) cervical spine

The NASS consists of two subscales: 1) pain and disability (11 questions) and 2) neurogenic symptoms (8 questions). Subscale pain and disability addresses perceived impairment in everyday life (e.g. during dressing, walking, sleeping), at work (e.g. during lifting, sitting, writing), or during leisure activities (e.g. while travelling) [29]. Subscale neurogenic symptoms addresses feelings of weakness, numbness, or pins and needles in the upper limb. All items refer to perception over the last seven days and can be judged on a scale from one to six (e.g. level one: “I can perform without pain.” to level six: “Due to my pain level I cannot perform at all.”). A high score indicates a high degree of impairment [31]. A change of one point is considered to be clinically relevant [13]. The NASS has been officially translated into German showing a very good reliability for both subscales (0.90 and 0.89) [13,14] and have been used in WAD patients [15,16]. The total score of subscale pain and disability (11 questions) was used to test the concurrent validity with the WDQ-G.

##### The Medical Outcomes Study (MOS) 36-item short form health survey (SF-36)

The SF-36 serves to determine perceived general health and quality of life [32]. Worldwide it is the most extensively used multidimensional questionnaire evaluating general health state containing 36 items clustered in two components: 1) physical health and 2) mental health with four multi-item scales each. Physical health contains physical function (10 items), role physical (4 items), bodily pain (2 items), and general health (5 items). Mental health includes mental health (5 items), role emotional (3 items), social function (2 items), vitality (4 items) and change in health (1 item). Item scores for each dimension are coded, summed and transformed to a scale from 0 (worst possible health state measured by the questionnaire) to 100 (best possible health sate). The higher value indicates a better evaluation of health. During the International Quality of Life Assessment (IQOLA) Project the SF-36 has been translated according to international guidelines into more than 40 different languages [21]. It has been used in more than 30 different disease conditions including patients with migraine, with pain in the upper or lower back, WAD, osteoarthritis or joint replacements [17-21,33-35]. Subscale bodily pain was used to further test the WDQ-G for concurrent validity. Both items ask about the extent of pain and its interference with the individual’s work capability.

Additionally, participants were asked to rate their actual subjective pain intensity on a Visual Analogue Scale (VAS). Pain is indicated on a horizontal 10-cm straight line anchored by two extremes of pain: “no pain” and “pain as bad as it could be” [31].

Anonymised and completed SF-36 and NASS questionnaires were scanned to upload by secure data transfer to an independent company (RehabNET AG, Zurich, Switzerland) for data assembly and subsequently returned for in-house analysis. Questionnaires for demographic and descriptive statistics as well as VAS and WDQ data were recorded manually within the clinic using Microsoft Excel 2003. All data were eventually assembled for statistical examination with the Statistical Package for Social Sciences (SPSS).

#### Participants

Patients referred to inpatient rehabilitation were asked to participate when they fulfilled the following selection criteria: German speaking females and males with an acceleration-deceleration event of the head with or without a mild traumatic brain injury (MTBI), being older than 18 years, understand the aim and procedure of the study, and given written informed consent. Patients were excluded if they had additional neurological or psychiatric diseases, if they needed supporting devices for walking, e.g. walking sticks, or if they had additional systemic diseases, e.g. Fibromyalgia.

### Statistical analyses

Patient and questionnaire descriptive data were calculated representing frequencies, means and standard deviations or confidence intervals. Concurrent validity was estimated by computing the Pearson Product–Moment Correlation Coefficient (r) between WDQ-G total score and subscale pain and disability of the NASS questionnaire, and between WDQ-G total score and subscale bodily pain of the SF-36. Internal consistency was computed using data from the first measurement event at study entry. Additionally, the inter-item correlation matrix was observed to detect very high correlation indicating item redundancy. All analyses were performed with SPSS version 16, 2007 (SPSS, Inc., Chicago Ill) with p ≤ 0.05.

## Results

### First part: translation and adaptation process

During the translation stages 1 (forward) and 3 (backward) three different German and English versions were produced. In particular, the wording of the first parts of the questions on “How much do your whiplash symptoms interfere…” or “How much pain/ sadness/anger/ do you…” was not congruently translated. The synthesis (stage 2) was necessary to agree on a sole German version providing the basis for the backward translations conducted by three independent translators. During the consensus conference (stage 4) the wordings at the beginning of questions 1, 3, 6, 10, 11, and 12 were again modified to avoid the implication that patients have to have pain after whiplash injury, and if so that it should be on a high level, e.g. question 3: the original WDQ asks: “How much do your whiplash symptoms interfere…”. For the German WDQ this had to be adapted to: “To what extent do your whiplash symptoms impair…”. Furthermore, the scale descriptions on the lower and higher ends were shortened to minimise ambiguity.

In agreement with the authors of the original Australian WDQ it was decided to modify the description on how to fill in the questionnaire for two reasons: 1) to emphasise the scale (zero to ten) and 2) to minimise the risk that patients miss out items.

Original Australian version: “Please circle a number in each section to indicate how you have been affected by the whiplash injury and symptoms. If one or more questions are not relevant to you, please leave that section blank.”

Agreed German version: “For each question please circle on a scale from 0 to 10 the number corresponding to the extent to which you are affected by your whiplash symptoms. If one or more questions are not relevant, please cross them out”.

#### Pre-study patient survey

Only 47 of 60 randomly selected patients could be contacted by postal mail for the pre-study patient survey. The response rate was 31% representing 16 patients (age 46.8 ± 10.5 years, time since injury 6.4 ± 2.6 years, 13 females), who filled in the preliminary WDQ-G. Clarity of title 9.6 (± 0.9), instructions 9.3 (± 1.4), and questions 9.6 (± 0.7), as well as comprehensiveness of title 9.6 (± 0.7), instructions 9.3 (± 1.4), and questions 9.8 (± 0.4) was rated by 15 patients. Time needed to fill in the WDQ-G was 13.7 (± 9.0) minutes ranging from 1.2 to 30.0 minutes as reported by 14 patients. WDQ-G total score of all 16 patients was 69.4 (± 24.0) ranging from 6.0 to 103.0 points.

The participating teenagers had no problems in understanding the content and aim of the questionnaire, wording of all questions and scale descriptions.

### Second part: patient validation study

#### Patient validation study

The validation study was conducted between June 2006 and September 2008 in a midsize rehabilitation centre in the German-speaking part of Switzerland. During this time period subsequently referred patients (159) were screened for study eligibility. Exclusion reasons for 81 patients were: additional psychiatric (N = 9), neurological (N = 2), or systemic (N = 2) disease, insufficient language skills (N = 16), incomplete questionnaire return for first measurement event (N = 8). Further reasons for exclusion were (N = 52): necessity of walking aids, no written consent, early discharge, recent bone fracture, no WAD, and arthroscopy of the knee.

Eventually, a consecutive sample of 70 WAD patients (47 females, mean age = 43.4 ± 12.5 years, ranging from 21 to 75 years) could be recruited. Average time since injury was 1.5 ± 2.6 years on average (median 31 weeks, range 3.0 weeks to 17.8 years). Table 1 provides an overview on all questionnaire mean values at entry.

**Table 1:** Overview on pain, WDQ-G, NASS, and SF-36 data at entry

**Questionnaires**	**Subscales**	**N**	**Entry**				
			**Median**	**Mean**	**SD**	**Min**	**Max**
**Pain (VAS)**		67	6.2	6.0	1.9	1	9
**WDQ-G**		70	75.5	74.4	21.2	15	117
**NASS**	Pain and disability	68	3.5	3.5	0.8	1	5
	Neurogenic symptoms	67	2.3	2.5	1.3	1	5
**SF-36**	Physical functioning	69	65.0	60.0	23.6	10	100
	Physical role	62	0.0	13.7	28.5	0	100
	Bodily pain	69	22.0	25.7	15.4	0	84
	General health	67	50.0	53.0	17.6	20	100
	Vitality	68	30.0	30.7	21.4	0	95
	Social function	68	50.0	47.1	28.6	0	100
	Emotional role	62	66.7	57.0	46.1	0	100
	Mental health	68	60.0	58.6	23.2	8	100

**Legend:** N = number of patients, VAS = visual analogue scale, WDQ-G = German version of the Whiplash Disability Questionnaire, NASS = North American Spine Society, SF-35 = 36-Item Short Form Health Survey, SD = standard deviation, Min/Max = minimum and maximum of observed patient values.

#### WDQ-G responses

**Figure 1:**
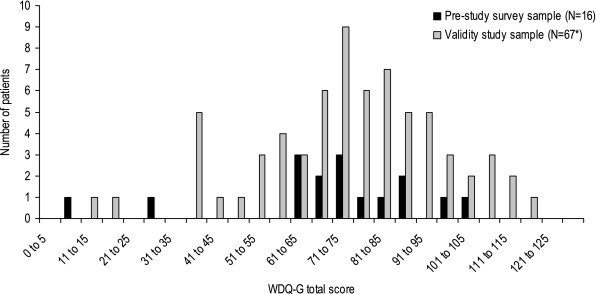
**Distribution of WDQ-G total scores.** Legend: N = sample size, WDQ-G = German version of the Whiplash Disability Questionnaire, * = three of 70 patients did not respond to all items of the questionnaire.

Figure 1 presents the number of responses for each WDQ-G question of the pre-study survey sample and the validation study sample. The WDQ-G mean total score of the pre-study survey sample was 69.4 (± 24.0) for 16 patients and 74.0 (± 21.3) for the inpatient validation study sample for 67 patients, respectively. Time needed to fill in the WDQ-G was 13.7 (± 9.0) minutes for the pre-study survey sample with data from 14 patients and 6.7 (± 3.4) minutes for the validation study sample with data from 22 patients only. Mean values for each of the 13 WDQ-G questions are presented in Table 2.

**Table 2:** Overview on WDQ-G mean scores for all 13 items

**Item**	**Mean (N = 67)**	**SD**	**Median**
1	6.3	1.9	7.0
2	2.3	2.1	2.0
3	7.2	2.2	8.0
4	4.6	2.6	5.0
5	6.1	2.5	7.0
6	7.2	2.5	7.0
7	6.5	2.2	7.0
8	7.9	2.3	8.0
9	6.7	2.5	7.3
10	5.1	2.7	5.0
11	4.4	3.0	5.0
12	4.2	3.1	4.0
13	6.3	2.7	7.0

**Legend:** N = sample size, SD = standard deviation.

#### Internal consistency

Based on the inter-item correlation, internal consistency (Cronbach’s α) of the German WDQ was α = 0.894 (N = 67). Similar to Pinfold *et al.*, no redundancy was found when considering inter-item correlation, which did not exceed 0.75 [8]. Observation of the item-total correlation revealed three low correlations: item 2 (personal care, r = 0.31), item 5 (sleep, r = 0.49), and item 11 (anger, r = 0.49). Calculating α without item 2 (α = 0.897), item 5 (α = 0.891) or item 11 (α = 0.893) did not interfere overall Cronbach’s alpha with α = 0.894 (13 items).

#### Concurrent validity

In relation to the second study aim, concurrent validity of the WDQ-G was determined with the subscale pain and disability of the NASS questionnaire (r = 0.74) and with the bodily pain subscale of the SF-36 (r = 0.71). Both correlations were determined highly significant with p < 0.01. Table 3 provides an overview on correlations of the WDQ-G with all questionnaire subscales (NASS and SF-36).

**Table 3:** WDQ-G correlation with the NASS, SF-36 and VAS pain at study entry

	**Subscale**	**Pearson’s r (N)**
Concurrent validity related subscales	NASS: Pain and disability	0.74** (68)
	SF36 Bodily pain	−0.71** (69)
	VAS	0.61** (70)
Further subscale correlations for informative purposes	SF36 Mental health	−0.69** (69)
	SF36 Physical function	−0.64** (69)
	SF36 Social function	−0.62** (69)
	SF36 Role emotional	−0.61** (69)
	SF36 Vitality: Entry	−0.54** (69)
	SF36 Physical role	−0.54** (65)
	SF36 General health	−0.27* (67)
	NASS: Neurogenic symptoms	0.43** (67)

**Legend:** N = sample size, VAS = visual analogue scale, NASS = North American Spine Society questionnaire, SF-36 = Medical Outcomes Study (MOS) 36-Item Short Form Health Survey, *p < 0.05, **p < 0.01 (two-tailed).

## Discussion

The study described a guideline-driven German translation, cross-cultural adaptation and validation process of a disease-specific questionnaire for WAD patients: the Whiplash Disability Questionnaire. In six predefined stages the Australian-based WDQ was forward and backward translated, approved by the original authors, evaluated by WAD patients, and tested for its quality criteria. As hypothesised, the WDQ-G correlated highly significant with the NASS subscale pain and disability and the SF-36 subscale bodily pain showing a good concurrent validity. Furthermore, the WDQ-G presents a high internal consistency. As a further development of the NDI, the WDQ covers specific aspects of impairment for WAD patients: role performance, tiredness, social and leisure (sporting and non-sporting) activity, emotional and cognitive impairments that can be evaluated on an eleven point rating scale [8].

In clinical trials, treatment effects and calculated effect sizes as well as recommended treatment guidelines are based on subjective and objective outcome measures. Those outcome measures are vital elements in the trial methodology. Therefore, it is essential to translate outcome measures in a standardised way into different languages to remain the original construct assessed and adopt it to the target country specific language, traditions, and customs. Furthermore, it is crucial to evaluate the quality criteria of the translated and adapted measurement [20,22]. The authors are confident that a rigorous process was applied to reach equivalence between the original WDQ and the resulting German version of the WDQ providing an assessment for use in clinical practice and research, which is supported by the excellent Cronbach’s α of 0.894.

Difference for time needed to fill in the questionnaire between pre-study sample (13.7 minutes) and the validation study sample (6.7 minutes) could be explained by the additional task assigned to the pre-study sample to also evaluate the WDQ-G’s clarity and comprehensiveness, whereas inpatients in the validation study sample only had to fill in the questionnaire.

The present patient validation sample showed a different gender distribution of 1.4:1 (female:male) compared to the patient sample in Pinfold *et al.*’s study with 4.3:1 [8] but similar gender distribution as indicated in the systematic efficacy review of Drescher *et al.* ranging between 1:1 to 2:1 [36]. Generally, the consecutive patient sample of the present validation study covers an older patient age range (>65) but can be compared with previous studies evaluating the use of the WDQ [8,30,37] or WAD interventions [36,38]. Demographic variables are also comparable with other German-speaking Swiss WAD inpatients [15].

Patients in the validation study showed the highest score for item 8 (7.89, sporting activity) and the lowest for item 2 (2.30, personal care). The average score for all 13 questions was 74.4. Those scores are almost identical to the results from Pinfold *et al.*[8]. However, for the English version, item 8 was scored lower (6.1) and the mean WDQ score was 55.7. Both differences could be attributed to the shorter time since injury onset in the present study (20 months vs. 48 months on average in Pinfold’s study). The scoring of item 2 and item 8 in the present study also suggest that there are no floor or ceiling effects.

The translation and cross-cultural adaptation process followed the guidelines proposed by Beaton *et al.*[22]. Stage 2 and 4 were essential to synthesise all produced forward and backward questionnaire translations. All produced variations to formulate title, questionnaire items, questionnaire and scale descriptions, and their meaning had to be considered. Adaptations in the formulation at the beginning of questions 1, 3, 6, 10, 11, and 12 of the scale descriptions emphasise the need for a standardised translation process. Adaptations were necessary to avoid the implication that patients have to have pain after whiplash injury and if so that it should be on a high level. The authors assume that the reformulation of the questions mentioned above do not have an influence on the construct under investigation since the sense of the questions remained unchanged. That could be demonstrated by the calculated Cronbach’s α (WDQ-G α = 0.89), which is only slightly lower than the original Australian version (WDQ α = 0.96) [8]. Furthermore, adaptations made were approved by all translators at the consensus conference and by the original authors of the Australian WDQ when reviewing all forward and backward translation documents.

To answer question four (driving or using public transport) patients differentiated between being the driver, the co-driver, or a passenger in a public transport vehicle. All three alternatives could be impaired on different severity levels after a whiplash injury. If a patient in the present validation study raised the question, which aspect should be evaluated, they were asked to indicate the impairment level for the most unpleasant situation. It is assumed that the differentiation can occur in all patients filling in the WDQ independent from different languages. Therefore, further research is needed to define a more precise patient instruction or add further questions to evaluate all three alternatives separately.

For a trustworthy questionnaire use in clinical routine or research it is important to determine quality criteria of the instrument including validity, reliability, and responsiveness. The present study focussed on the standardised translation process and data collection to determine concurrent validity and internal consistency. Meanwhile the paper was published.

### Study limitations

The presented study aimed to produce a robust German version of the WDQ by following strict guidelines published by Beaton *et al.*[22]. However, different recommendations exist on how to cross-culturally translate and adapt self-administered measurements. In the present study the team followed the forward-backward translation approach rather than the two panel approach suggested by McKenna *et al.*[39]. The two panel approach prefers expert and lay committee meetings and does not include a backward translation. In a randomised study on the two translation approaches applied to the Rheumatoid Arthritis Quality of Life (RAQoL) for Sweden none of the translated questionnaires was preferred by bilinguals [40]. Reliability and validity characteristics were similar in both RAQoL versions. However, in the present six stages WDQ translation and cross-cultural adaptation process, the backward translation and the consensus conference with multidisciplinary health professionals and language experts ensured a comprehensive and trustworthy German version.

In general, validity tests for self-administered questionnaires are difficult to implement and to compare with a gold standard, in particular as there is no gold standard for WAD. In the case of the present investigation, it was chosen to determine concurrent validity with the subscale pain and disability (NASS) and subscale bodily pain (SF-36) to approach a close conceptional association to established related questionnaires.

In the validation study, data from 70 patients was analysed to determine validity. It could be argued that the sample size was too small for a final validity analysis. However, in other publications on translation and validity studies sample sizes varied distinctly [19,41-43]. Recent publications provide suggested aids to the decision-making process on sample sizes for reliability and validity studies [44,45]. Hobart *et al.* suggest a sample of 20 for reliability studies and a sample of 80 or more for validation studies in neurology [44]. Javali *et al.* proposed a sample size of 50 to determine reliability for measures with a five-point Likert scale [45]. So far, no consensus has been reached on the ideal sample size. Apart of scientific reasoning, available financial and personnel resources have to be considered too.

Sample size was also the limiting factor to conduct factor analysis. It is recommended to have a case:item ratio of 10:1 requiring at least 130 cases for a WDQ-G factor analysis [46]. In some circumstances a sample size of 100 cases might be sufficient, nevertheless, in the present study only a sample of 70 cases could be recruited [46]. The authors of the original Australian WDQ performed a factor analysis with 101 cases and confirmed the unifactorial structure of the WDQ [8]. For now it must be assumed that the rigorous cross-cultural translation and adaptation process based on international guidelines resulted in a German WDQ with good concurrent validity, internal consistency, and a similar questionnaire structure as the Australian original version.

## Conclusion

The WDQ-G is a self-administered disease-specific outcome measure showing a high internal consistency and good concurrent validity. After the official translation and cross-cultural adaptation process the WDQ-G represents preliminary validity evidence and can be used with German-speaking WAD patients to evaluate patients’ impairments in different domains: activities of daily living, career, leisure activities, social life, and care for others. Further research should include a factor analysis to confirm the unifactorial structure of the questionnaire.

The German version of the WDQ can be obtained free of charge from the first author: Dr. Corina Schuster: c.schuster@reha-rhf.ch.

## Abbreviations

α: Cronbach’s alpha; MTBI: Mild traumatic brain injury; N: Sample size; NASS: North American spine societies questionnaire for neck pain; NDI: Neck disability index; NPQ: Northwick park neck pain questionnaire; r: Pearson product–moment correlation coefficient; RAQoL: Rheumatoid arthritis quality of life questionnaire; SF-36: The medical outcomes study 36-item short form health survey; SPSS: Statistical package for social sciences; VAS: Visual analogue scale; WAD: Whiplash associated disorders; WDQ: Whiplash disability questionnaire; WDQ-G: German version of the whiplash disability questionnaire.

## Competing interests

The authors declare that they have no financial or non-financial competing interests.

The preliminary results have been presented as a poster at the 5^th^ Word Conference on NeuroRehabilitation in Brasilia 2008 and at the 16th World Congress for Physical Therapy in Amsterdam 2011.

## Authors’ contributions

CS was the project leader. She wrote the study protocol and was involved in the translation and validation process, data collection, analyses and interpretation, and manuscript writing. MM was involved in data analysis and interpretation, and he critically reviewed earlier versions of the manuscript. TE was involved in study design, data interpretation and he critically revised the manuscript. All authors gave final approval of the manuscript.

## References

[B1] Guzman J Haldeman S Carroll LJ Carragee EJ Hurwitz EL Peloso P Nordin M Cassidy JD Holm LW Cote P Clinical practice implications of the Bone and Joint Decade 2000–2010 Task Force on Neck Pain and Its Associated Disorders: from concepts and findings to recommendations Spine 2008 33 4 Suppl S199 213 18204393 10.1097/BRS.0b013e3181644641

[B2] Spitzer WO Skovron ML Salmi LR Cassidy JD Duranceau J Suissa S Zeiss E Scientific monograph of the Quebec Task Force on Whiplash-Associated Disorders: redefining “whiplash” and its management Spine 1995 20 8 Suppl 1S 73S 7604354

[B3] Holm LW Carroll LJ Cassidy JD Hogg-Johnson S Cote P Guzman J Peloso P Nordin M Hurwitz E van der Velde G The burden and determinants of neck pain in whiplash-associated disorders after traffic collisions: results of the Bone and Joint Decade 2000–2010 Task Force on Neck Pain and Its Associated Disorders Spine 2008 33 4 Suppl S52 59 18204401 10.1097/BRS.0b013e3181643ece

[B4] Guzman J Hurwitz EL Carroll LJ Haldeman S Cote P Carragee EJ Peloso PM van der Velde G Holm LW Hogg-Johnson S A new conceptual model of neck pain: linking onset, course, and care: the Bone and Joint Decade 2000–2010 Task Force on Neck Pain and Its Associated Disorders Spine 2008 33 4 Suppl S14 23 18204387 10.1097/BRS.0b013e3181643efb

[B5] Hains F Waalen J Mior S Psychometric properties of the neck disability index J Manipulative Physiol Ther 1998 21 75 80 9502061

[B6] Leak AM Cooper J Dyer S Williams KA Turner-Stokes L Frank AO The Northwick Park Neck Pain Questionnaire, devised to measure neck pain and disability Br J Rheumatol 1994 33 5 469 474 10.1093/rheumatology/33.5.469 8173853

[B7] Hoving JL O’Leary EF Niere KR Green S Buchbinder R Validity of the neck disability index, Northwick Park neck pain questionnaire, and problem elicitation technique for measuring disability associated with whiplash-associated disorders Pain 2003 102 3 273 281 10.1016/S0304-3959(02)00406-2 12670669

[B8] Pinfold M Niere KR O’Leary EF Hoving JL Green S Buchbinder R Validity and internal consistency of a whiplash-specific disability measure Spine 2004 29 3 263 268 10.1097/01.BRS.0000107238.15526.4C 14752347

[B9] Schellingerhout JM Heymans MW Verhagen AP de Vet HC Koes BW Terwee CB Measurement properties of translated versions of neck-specific questionnaires: a systematic review BMC Med Res Methodol 2011 11 87 10.1186/1471-2288-11-87 21645355 PMC3118950

[B10] Schellingerhout JM Verhagen AP Heymans MW Koes BW de Vet HC Terwee CB Measurement properties of disease-specific questionnaires in patients with neck pain: a systematic review Qual Life Res 2011 21 4 659 670 21735306 10.1007/s11136-011-9965-9 PMC3323817

[B11] Bremerich FH Grob D Dvorak J Mannion AF The Neck Pain and Disability Scale: cross-cultural adaptation into German and evaluation of its psychometric properties in chronic neck pain and C1-2 fusion patients Spine 2008 33 9 1018 1027 10.1097/BRS.0b013e31816c9107 18427324

[B12] Scherer M Blozik E Himmel W Laptinskaya D Kochen MM Herrmann-Lingen C Psychometric properties of a German version of the neck pain and disability scale Eur Spine J 2008 17 7 922 929 10.1007/s00586-008-0677-y 18437433 PMC2443271

[B13] Pose B Sangha O Peters A Wildner M Validation of the German version of the North American Spine Society (NASS) cervical and lumbar spine outcome instrument Zeitschrift fur Orthopadie und ihre Grenzgebiete 1999 137 5 437 441 10.1055/s-2008-1037387 10549122

[B14] Sangha O Wildner M Peters A Evaluation of the North American Spine Society Instrument for assessment of health status in patients with chronic backache Z Orthop Ihre Grenzgeb 2000 138 5 447 451 10.1055/s-2000-10176 11084747

[B15] Angst F Verra ML Lehmann S Gysi F Benz T Aeschlimann A Responsiveness of the cervical Northern American Spine Society questionnaire (NASS) and the Short Form 36 (SF-36) in chronic whiplash Clin Rehabil 2012 26 2 142 151 10.1177/0269215511414158 21856722

[B16] Stoll T Huber E Bachmann S Baumeler HR Mariacher S Rutz M Schneider W Spring H Aeschlimann A Stucki G Validity and sensitivity to change of the NASS questionnaire for patients with cervical spine disorders Spine 2004 29 24 2851 2855 10.1097/01.brs.0000147802.57484.77 15599289

[B17] Ware JE Jr Gandek B Overview of the SF-36 Health Survey and the International Quality of Life Assessment (IQOLA) Project J Clin Epidemiol 1998 51 11 903 912 10.1016/S0895-4356(98)00081-X 9817107

[B18] Weigl M Ewert T Kleinschmidt J Stucki G Measuring the outcome of health resort programs J Rheumatol 2006 33 4 764 770 16583479

[B19] Bullinger M German translation and psychometric testing of the SF-36 Health Survey: preliminary results from the IQOLA Project. International Quality of Life Assessment Soc Sci Med 1995 41 1359 1366 10.1016/0277-9536(95)00115-N 8560303

[B20] Bullinger M Alonso J Apolone G Leplege A Sullivan M Wood-Dauphinee S Gandek B Wagner A Aaronson N Bech P Translating health status questionnaires and evaluating their quality: the IQOLA Project approach. International Quality of Life Assessment J Clin Epidemiol 1998 51 11 913 923 10.1016/S0895-4356(98)00082-1 9817108

[B21] Ware JE SF-36® Health Survey Updatehttp://www.sf-36.org/tools/sf36.shtml (access 28122012)23912853

[B22] Beaton DE Bombardier C Guillemin F Ferraz MB Guidelines for the process of cross-cultural adaptation of self-report measures Spine 2000 25 24 3186 3191 10.1097/00007632-200012150-00014 11124735

[B23] Frinking E Kahan JP van het Loo M Vader J-P Association SI Risk profiles and appropriate treatment therapies for whiplash associated disorders. Volume 19 2003 RAND Europe

[B24] Stoll T Gordon C Seifert B Richardson K Malik J Bacon PA Isenberg DA Consistency and validity of patient administered assessment of quality of life by the MOS SF-36; its association with disease activity and damage in patients with systemic lupus erythematosus J Rheumatol 1997 24 8 1608 1614 9263159

[B25] Bullinger M German translation and psychometric testing of the SF-36 Health Survey: preliminary results from the IQOLA Project International Quality of Life Assessment Soc Sci Med 1995 41 10 1359 1366 10.1016/0277-9536(95)00115-N 8560303

[B26] Ware JE Jr Kosinski M Gandek B Aaronson NK Apolone G Bech P Brazier J Bullinger M Kaasa S Leplege A The factor structure of the SF-36 Health Survey in 10 countries: results from the IQOLA Project International Quality of Life Assessment. J Clin Epidemiol 1998 51 11 1159 1165 10.1016/s0895-4356(98)00107-3 9817133

[B27] Turk DC Rudy TE Salovey P The McGill Pain Questionnaire reconsidered: confirming the factor structure and examining appropriate uses Pain 1985 21 4 385 397 10.1016/0304-3959(85)90167-8 4000688

[B28] Kiss IC Müller H Abel M The McGill pain questionnaire — German version. A study on cancer pain Pain 1987 29 2 195 207 10.1016/0304-3959(87)91036-0 2886967

[B29] Daltroy LH Cats-Baril WL Katz JN Fossel AH Liang MH The North American spine society lumbar spine outcome assessment Instrument: reliability and validity tests Spine 1996 21 6 741 749 10.1097/00007632-199603150-00017 8882698

[B30] Niere K The Whiplash Disability Questionnaire (WDQ) Aust J Physiother 2006 52 2 151 151 10.1016/S0004-9514(06)70053-8 16805043

[B31] Finch E Brooks D Stratford P Physical Rehabilitation Outcome Measures: A Guide to Enhanced Clinical Decision Making 2002 2 Hamilton, Ontario: Canadian Physiotherapy Association

[B32] Ware JE Jr Sherbourne CD The MOS 36-item short-form health survey (SF-36) I. Conceptual framework and item selection Med Care 1992 30 6 473 483 10.1097/00005650-199206000-00002 1593914

[B33] Stoll T Gordon C Seifert B Richardson K Malik J Bacon PA Isenberg DA Consistency and validity of patient administered assessment of quality of life by the MOS SF-36; its association with disease activity and damage in patients with systemic lupus erythematosus J Rheumatol 1997 24 1608 1614 9263159

[B34] Stucki G Liang MH Phillips C Katz JN The Short Form-36 is preferable to the SIP as a generic health status measure in patients undergoing elective total hip arthroplasty Arthritis Care Res 1995 8 174 181 10.1002/art.1790080310 7654802

[B35] Angst F Aeschlimann A Steiner W Stucki G Responsiveness of the WOMAC osteoarthritis index as compared with the SF-36 in patients with osteoarthritis of the legs undergoing a comprehensive rehabilitation intervention Ann Rheum Dis 2001 60 834 840 11502609 PMC1753825

[B36] Drescher K Hardy S Maclean J Schindler M Scott K Harris SR Efficacy of postural and neck-stabilization exercises for persons with acute whiplash-associated disorders: a systematic review Physiother Can 2008 60 3 215 223 10.3138/physio.60.3.215 20145754 PMC2792777

[B37] Willis C Niere KR Hoving JL Green S O’Leary EF Buchbinder R Reproducibility and responsiveness of the Whiplash Disability Questionnaire Pain 2004 110 3 681 688 10.1016/j.pain.2004.05.008 15288409

[B38] Cote P Cassidy JD Carroll L Frank JW Bombardier C A systematic review of the prognosis of acute whiplash and a new conceptual framework to synthesize the literature Spine 2001 26 19 E445 458 10.1097/00007632-200110010-00020 11698904

[B39] McKenna SP Doward LC Meads D Patrick D Tennant A Summary of Needs-Based Quality of Life Instruments Value Health 2004 7 S39 S40 10.1111/j.1524-4733.2004.7s109.x 15367243

[B40] Hagell P Hedin PJ Meads DM Nyberg L McKenna SP Effects of method of translation of patient-reported health outcome questionnaires: a randomized study of the translation of the Rheumatoid Arthritis Quality of Life (RAQoL) Instrument for Sweden Value Health 2010 13 4 424 430 10.1111/j.1524-4733.2009.00677.x 20070642

[B41] Stucki G Meier D Stucki S Michel BA Tyndall AG Dick W Theiler R Evaluation of a German version of WOMAC (Western Ontario and McMaster Universities) Arthrosis Index Z Rheumatol 1996 55 40 49 8868149

[B42] Offenbacher M Ewert T Sangha O Stucki G Validation of a German version of the ‘Disabilities of Arm, Shoulder and Hand’ questionnaire (DASH-G) Z Rheumatol 2003 62 168 177 10.1007/s00393-003-0461-7 12721705

[B43] Offenbaecher M Waltz M Schoeps P Validation of a German version of the Fibromyalgia Impact Questionnaire (FIQ-G) J Rheumatol 2000 27 8 1984 1988 10955342

[B44] Hobart J Cano S Warner T Thompson A What sample sizes for reliability and validity studies in neurology? J Neurol 2012 259 12 2681 2694 10.1007/s00415-012-6570-y 22729386

[B45] Javali SB Gudaganavar NV Raj SM Effect of varying sample size in estimation of reliability coefficients of internal consistency WebmedCentral BIOSTATISTICS 2011 2 2 WMC001572

[B46] Munro BH Statistical methods for health care research 2005 5 Philadelphia, Penn.; London: Lippincott Williams & Wilkins

